# Principles of Military Surgery

**Published:** 1830-04-01

**Authors:** 


### VIII.

PRINCIPLES OF MILITARY SURGERY, COMPRISING OBSERVATIONS ON THE ARRANGEMENT, POLICE, AND PRACTICE OF HOSPITALS, AND ON THE HISTORY, TREATMENT, AND ANOMALIES OF VARIOLA AND SYPHILIS. Illustrated with Cases and Dissections. By *John Hennen*, M.D. F.R.S. E. Inspector of Military Hospitals. Third Edition. With Life of the Author, by his Son, Dr. *John Hennen*. London, John Wilson, Princes Street, Soho. Octavo, pp. 583.

WE have much pleasure in announcing a third edition of the late Dr. Hennen's able work on Military Surgery. As we have already fully reviewed a former edition, and the work is so well known and appreciated, we have little to do now but to notice any additions or corrections that may have been made.

The alterations are not very material—references to works are rather more numerous—there are also a few additional observations, and the plates, which increased the expense of the second edition, have been omitted —instead of which we have a brief but interesting biographical notice of the author. And, as it may prove interesting to many of our readers to trace the career of one of the most distinguished ornaments of military surgery, we will make a slight sketch from the materials his son has placed before us.

Dr. Hennen was born at Castlebar, County Mayo, Ireland, and passed the first eighteen years of his life in his native town, and studied medicine under his father, who was a practitioner of some eminence. During part of this time he was dresser at the County Infirmary—in due time he proceeded to Edinburgh, and studied under the second Monro and Dr. Black. In 1798 he received his diploma from the College of Surgeons of that city, and in 1800 was appointed assistant-surgeon to the 40th regiment of foot: he was afterwards removed to the 3d dragoons—and after serving in various parts of the Mediterranean, in Ireland, Scotland, &c. in 1809, he proceeded to Portugal, where his unwearied zeal attracted the attention of the head of the medical department, Sir J. Macgregor—and in 1811 he was promoted to the rank of surgeon to the forces, and had the charge of some of the most important surgical hospitals in the Peninsula. In the peace in 1814 he retired to Dumfries, and practised for a short time—in 1815 he was again called into action, and after the battle of Waterloo he had the sole superintendance of the wounded General Staff. After performing duty at Portsmouth, &c., he was removed to Edinburgh in 1817, where he published his first edition of the work before us. In the Winter of 1820, he became a lecturer on Military Surgery, and in the same year published a second edition. In 1821 he was ordered to the Mediterranean, and finally was placed in charge of the Medical department of Gibraltar in 1826, where he terminated his arduous career, after thirty one years spent in active employment and devoted to the public service. In October, 1828, he was attacked with the yellow fever, of which he died early in the next month—and as a proof of his philanthropic zeal, it is stated that he was acting in the discharge of his duties up to a few hours of his death.

A monument has been erected by subscription to his memory in Gibraltar, as a mark of gratitude for his indefatigable exertions in the service of the inhabitants.

In conclusion, it is almost unnecessary to recommend the work to the attention of every young practitioner, but more especially to those entering the army—it is a work full of sound chirurgical knowledge—and abounds in the information which is most required by the inexperienced army surgeon.

